# Long non-coding RNA CDKN2B-AS1 enhances LPS-induced apoptotic and inflammatory damages in human lung epithelial cells via regulating the miR-140-5p/TGFBR2/Smad3 signal network

**DOI:** 10.1186/s12890-021-01561-z

**Published:** 2021-06-14

**Authors:** Bing Wang, Qi Sun, Wen Ye, Lianghai Li, Ping Jin

**Affiliations:** 1 Department of Intensive Care Unit, The Second Clinical Medical College, Jingzhou Central Hospital, Yangtze University, No. 1 Renmin Road, Jingzhou District, Jingzhou, 434000 Hubei China; 2 Blood Purification Center, The Second Clinical Medical College, Jingzhou Central Hospital, Yangtze University, Jingzhou, 434000 Hubei China

**Keywords:** CDKN2B-AS1, miR-140-5p, Acute lung injury, Sepsis, TGFBR2, Smad3

## Abstract

**Background:**

Sepsis is a complicated disease with systemic inflammation or organ dysfunction, and it is the leading cause of acute lung injury (ALI). Long non-coding RNAs (lncRNAs) have played important roles in the pathogenesis of sepsis. This study was designed to explore the biological function and regulatory mechanism of cyclin-dependent kinase inhibitor 2B antisense RNA 1 (CDKN2B-AS1) in lipopolysaccharide (LPS)-induced lung injury.

**Methods:**

ALI model was established after human lung epithelial cell line BEAS-2B was exposed to LPS. CDKN2B-AS1, microRNA-140-5p (miR-140-5p) and transforming Growth Factor Beta Receptor II (TGFBR2) levels were detected by quantitative real-time polymerase chain reaction (qRT-PCR). Cell viability was measured using Cell Counting Kit-8 (CCK-8). Cell apoptosis was assessed by caspase3 activity and flow cytometry. Inflammatory cytokines were examined via enzyme-linked immunosorbent assay (ELISA). Protein analysis was performed through western blot. Dual-luciferase reporter, RNA immunoprecipitation (RIP) and pull-down assays were applied to validate the interaction between targets.

**Results:**

CDKN2B-AS1 and TGFBR2 were abnormally upregulated in sepsis patients. Functionally, CDKN2B-AS1 or TGFBR2 knockdown promoted cell growth but inhibited cell apoptosis and inflammatory response in LPS-treated BEAS-2B cells. Moreover, the regulation of CDKN2B-AS1 in LPS-induced cell injury was achieved by increasing the TGFBR2 expression. CDKN2B-AS1 was identified as a miR-140-5p sponge and TGFBR2 was a target of miR-140-5p. Furthermore, CDKN2B-AS1 could regulate the TGFBR2/Smad3 pathway by sponging miR-140-5p.

**Conclusions:**

These results suggested that CDKN2B-AS1 contributed to the LPS-mediated apoptosis and inflammation in BEAS-2B cells via the miR-140-5p/TGFBR2/Smad3 axis.

**Supplementary Information:**

The online version contains supplementary material available at 10.1186/s12890-021-01561-z.

## Background

Sepsis is a fatal systemic disease with high mortality and long-term morbidity, and it leads to the inflammatory response in many organs (such as lung and kidney) [1, 2]. Acute lung injury (ALI) is a common sepsis-related lung disease. The acute inflammation and tissue injury in lung can evoke the dysfunction of the alveolar epithelial membranes [3–5]. Lipopolysaccharide (LPS) is the main component of cell wall of Gram-negative bacteria, and it has been considered as a risk factor of cell inflammation in sepsis [6, 7]. Seeking the effective biomarker is essential to improve the treatment of sepsis-induced ALI.

Long-coding RNAs (lncRNAs) are common noncoding RNAs (ncRNAs) with regulatory function in human diseases [8]. LncRNA cyclin-dependent kinase inhibitor 2B antisense RNA 1 (CDKN2B-AS1) has been involved in the progression of various diseases, including osteosarcoma [9], atherosclerosis [10], coronary heart disease [11], and diabetic retinopathy [12]. CDKN2B-AS1 was also associated with non-cancerous lung diseases [13]. Gui et al. have found that CDKN2B-AS1 was highly expressed in sepsis patients and it was related to inflammatory injury [14]. The biological role of CDKN2B-AS1 remains to be explored in LPS-treated lung epithelial cells.

MicroRNAs (miRNAs) can regulate the transcriptional and post-transcriptional levels of downstream genes by targeting the 3′-untanlsated regions (3’UTRs) of messenger RNAs [15]. MicroRNA-140-5p (miR-140-5p) has participated in the pathogenic development of many human diseases. For example, Du et al. reported that miR-140-5p affected the progression of Hirschsprung’s disease via targeting EGR2 [16]. Wang et al. found that miR-140-5p inhibited neuroinflammation and brain injury in intracerebral hemorrhage by downregulating the expression of TLR4 [17]. In addition, miR-140-5p served as an anti-inflammatory factor in ALI via targeting TLR4 [18].

Transforming Growth Factor Beta Receptor II (TGFBR2) is one member of TGF-β signaling family, and it has been correlated to the inflammatory response through affecting the small mothers against decapentaplegic (Smad) pathway [19]. Cao et al. reported that miR-145 inhibited the sepsis-induced ALI via the direct downregulation of TGFBR2 [20]. Moreover, TGFBR2/Smad axis was involved in the LPS-induced inflammation in HUVECs [21].

LncRNAs can regulate the downstream genes by interacting with miRNAs [22]. Herein, we hypothesized that CDKN2B-AS1 acted as a sponge of miR-140-5p and miR-140-5p targeted TGFBR2. More importantly, we investigated the regulatory effect of CDKN2B-AS1 on the TGFBR2/Smad3 pathway by targeting miR-140-5p. This study focused on the functional role and regulatory mechanism of CDKN2B-AS1 in LPS-induced cell damages of lung epithelial cells.

## Methods

### Serum samples

The blood samples were collected from sepsis patients (n = 47) at Jingzhou Central Hospital. Patients were fasted before sample collection. Sepsis was diagnosed by two professional pathologists according to the Third International Consensus Definitions for Sepsis and Septic Shock (Sepsis-3) [23]. The healthy blood samples were acquired from healthy populations (n = 55) at the physical examination center of Jingzhou Central Hospital. These blood samples were centrifuged at 1200 rpm for 10 min, then the supernatant serums were preserved at − 80 °C. The informed consent forms were provided by all participants. The collection and use of patient samples were approved by the Ethics Committee of Jingzhou Central Hospital.

### Cell culture and LPS induction

Human lung epithelial cell line BEAS-2B was bought from American Type Culture Collection (ATCC, Manassas, VA, USA). Cells were cultured in Dulbecco’s modified eagle medium (DMEM; Hyclone, Logan, UT, USA) containing 10% fetal bovine serum (FBS; Gibco, Carlsbad, CA, USA), 100 U/mL penicillin and 1000 µg/mL streptomycin (Gibco) in a 37 °C, 5% CO_2_ humid incubator (Thermo Fisher Scientific, Waltham, MA, USA). BEAS-2B cells were treated with 1 µg/mL LPS (from *Escherichia coli*; catalogue number: L4391; Sigma-Aldrich, St. Louis, MO, USA) for 12 h [24].

### Cell transfection

4 × 10^3^ LPS-treated BEAS-2B cells were plated into the 96-well plates overnight. The 60%-confluent monolayer cells were transfected with small interfering RNA (siRNA) targeting CDKN2B-AS1 (si-CDKN2B-AS1#1, si-CDKN2B-AS1#2 and si-CDKN2B-AS1#3), siRNA targeting TGFBR2 (si-TGFBR2#1, si-TGFBR2#2 and si-TGFBR2#3), miR-140-5p mimic and inhibitor (miR-140-5p and anti-miR-140-5p), or the constructed pcDNA-TGFBR2 vector (TGFBR2) using Lipofectamine™ 3000 Transfection Reagent (Invitrogen, Carlsbad, CA, USA). The corresponding si-NC, miR-NC, anti-NC and pcDNA vector were used the negative controls. These oligonucleotides were purchased from RIBOBIO (Guangzhou, China) and the empty pcDNA vector was bought from Invitrogen.

### The quantitative real-time polymerase chain reaction (qRT-PCR)

Total RNA was extracted by TRI Reagent (Sigma-Aldrich) and the complementary DNA (cDNA) was synthesized by reverse transcription (1 µg RNA) using ReverTra Ace® qPCR RT Kit (Toyobo, Kita-Ku, Osaka, Japan). SYBR® Green Realtime PCR Master Mix (Toyobo) was used for expression detection. Data analysis was performed via the 2^−∆∆Ct^ method, with glyceraldehyde-phosphate dehydrogenase (GAPDH; for CDKN2B-AS1 and TGFBR2) and U6 (for miR-140-5p) as the housekeeping genes. The primers were shown in Table 1.

**Table 1:** Primer sequences used for qRT-PCR

Name	Primer sequences
CDKN2B-AS1	Forward: 5′-CACTGAGGCCCACACCTATT-3′ Reverse: 5′-TCCCTGCAGGAAAAATCATC-3′
miR-140-5p	Forward: 5′-TGCGGCAGTGGTTTTACCCTATG-3′ Reverse: 5′-CCAGTGCAGGGTCCGAGG − 3′
TGFBR2	Forward: 5′-GTAGCTCTGATGAGTGCAATGAC-3′ Reverse: 5’-CAGATATGGCAACTCCCAGTG-3’
GAPDH	Forward: 5′-GACCACAGTCCATGCCATCAC-3′ Reverse: 5′-ACGCCTGCTTCACCACCTT-3′
U6	Forward: 5′-CTCGCTTCGGCAGCACA-3′ Reverse: 5′-ACGCTTCACGAATTTGCGT-3′

### Cell counting Kit-8 (CCK-8) assay

1 × 10^4^ BEAS-2B cells were planted into the 96-well plates. Cell transfection was conducted at 37 °C for 48 h, followed by the incubation of CCK-8 solution (Dojindo, Kumamoto, Japan) with 10 µL/well (no bubbles). 2 h later, the absorbance at 450 nm was examined by the microplate reader (Thermo Fisher Scientific).

### Caspase3 activity detection

Caspase-3 Assay Kit-Colorimetric (Dojindo) was used for the detection of caspase3 activity. 4 × 10^6^ cells were washed with phosphate buffer solution (PBS; Hyclone) and lysed in 150 µL Lysis Buffer on the ice. Cell lysates were centrifuged at 10,000 ×*g* for 1 min, then the supernatant solution was transferred intro a new tube and protein concentration was determined by the Bradford method. The 96-well plates were added with 10 µL Substrate, 40 µL Assay Buffer and 50 µL cell samples (protein density of 1.0 µg/mL) for 2 h, then the absorbance was read at 450 nm using a microplate reader.

### Flow cytometry

6 × 10^4^ cells were collected for cell apoptosis detection through the double staining with Annexin V-fluorescein isothiocyanate (FITC) and propidium iodide (PI). The procedures were in accordance with the manufacturer’s specification of eBioscience™ Annexin V-FITC Apoptosis Detection Kit (Invitrogen) [25]. The apoptotic cells can be labeled by Annexin V (+)/PI (−) and Annexin V (+)/PI (+) on the flow cytometer (BD Biosciences, San Diego, CA, USA). The apoptotic rate was calculated using the formula: apoptotic cells/total cells × 100%.

### Enzyme-linked immunosorbent assay (ELISA)

The concentrations of Interleukin-1 (IL-1), Interleukin-6 (IL-6) and tumor necrosis factor-alpha (TNF-α) were examined using the corresponding ELISA Kits (Sigma-Aldrich), according to the user’s manuals. In addition, the protein level of TGFBR2 was detected by Human TGFBR2 ELISA Kit (Sigma-Aldrich).

### Western blot

Proteins were extracted by RIPA buffer (Millipore, Billerica, MA, USA) and the concentration was determined by BCA kit (Thermo Fisher Scientific). 40 µg proteins were loaded on the gels for western blot analysis as previously reported [26]. The primary antibodies against ki67 (#9129, 1:1000), cleaved-caspase3 (c-caspase3; #9664, 1:1000), NLR family pyrin domain containing 3 (NLRP3; #13,158, 1:1000), TGFBR2 (#79,424, 1:1000), phospho-Smad3 (#9520, 1:1000), GAPDH (#5174, 1:1000) and the secondary antibody Anti-rabbit IgG, HRP (#7074, 1:2000) were purchased from Cell Signaling Technology (CST, Boston, MA, USA). The protein bands were visualized using SignalFire™ Plus ECL Reagent (CST) and the expression level was analyzed using ImageLab software version 4.1 (Bio-Rad, Hercules, CA, USA). The original protein images were shown in Additional file 1.

### Dual-luciferase reporter assay

Luciferase reporter plasmids containing CDKN2B-AS1 or TGFBR2 sequence were constructed by cloning the sequence into the pGL-3 control vector (Promega, Madison, WI, USA). The wild-type (WT) and mutant-type (MUT) luciferase plasmids were named as WT-CDKN2B-AS1, WT-TGFBR2 3’UTR, MUT-CDKN2B-AS1, and MUT-TGFBR2 3’UTR. BEAS-2B cells were co-transfected with each plasmid and miR-140-5p or miR-NC, and the dual-luciferase reporter assay system (Promega) was used to measure the luciferase activity.

### RNA immunoprecipitation (RIP) assay

RIP assay was performed through Magna RIP RNA-Binding Protein Immunoprecipitation Kit (Millipore), according to the producer’s guidance [27]. Total RNA was isolated from the magnetic beads of Anti-IgG and Anti-Ago2 groups, followed by the expression analysis of CDKN2B-AS1 and miR-140-5p using qRT-PCR.

### Pull-down assay with biotin-coupled miR-140-5p

Biotin-coupled miR-140-5p or miR-NC (Bio-miR-140-5p, Bio-miR-NC) was purchased from RIBOBIO and transfected into BEAS-2B cells. Subsequently, cells were incubated with the streptavidin magnetic beads (Thermo Fisher Scientific) at 4 °C overnight. CDKN2B-AS1 level was determined via qRT-PCR after RNA purification.

### Statistical analysis

Our data were expressed by the mean ± standard deviation (SD). Statistical analysis was performed using SPSS 22.0 software. The linear relationship was analyzed using Pearson’s correlation coefficient. The data were analyzed by using Microsoft Excel Statistical Software (Jandel, San Rafael, CA) using *t* test for normally distributed data with equal variances. Difference analysis was performed using Student’s *t*-test for two groups and one-way analysis of variance (ANOVA) followed by Tukey’s test for multiple groups. *P* < 0.05 indicated a significant difference.

## Results

### CDKN2B-AS1 and TGFBR2 were overexpressed in sepsis patients

**Fig. 1:**
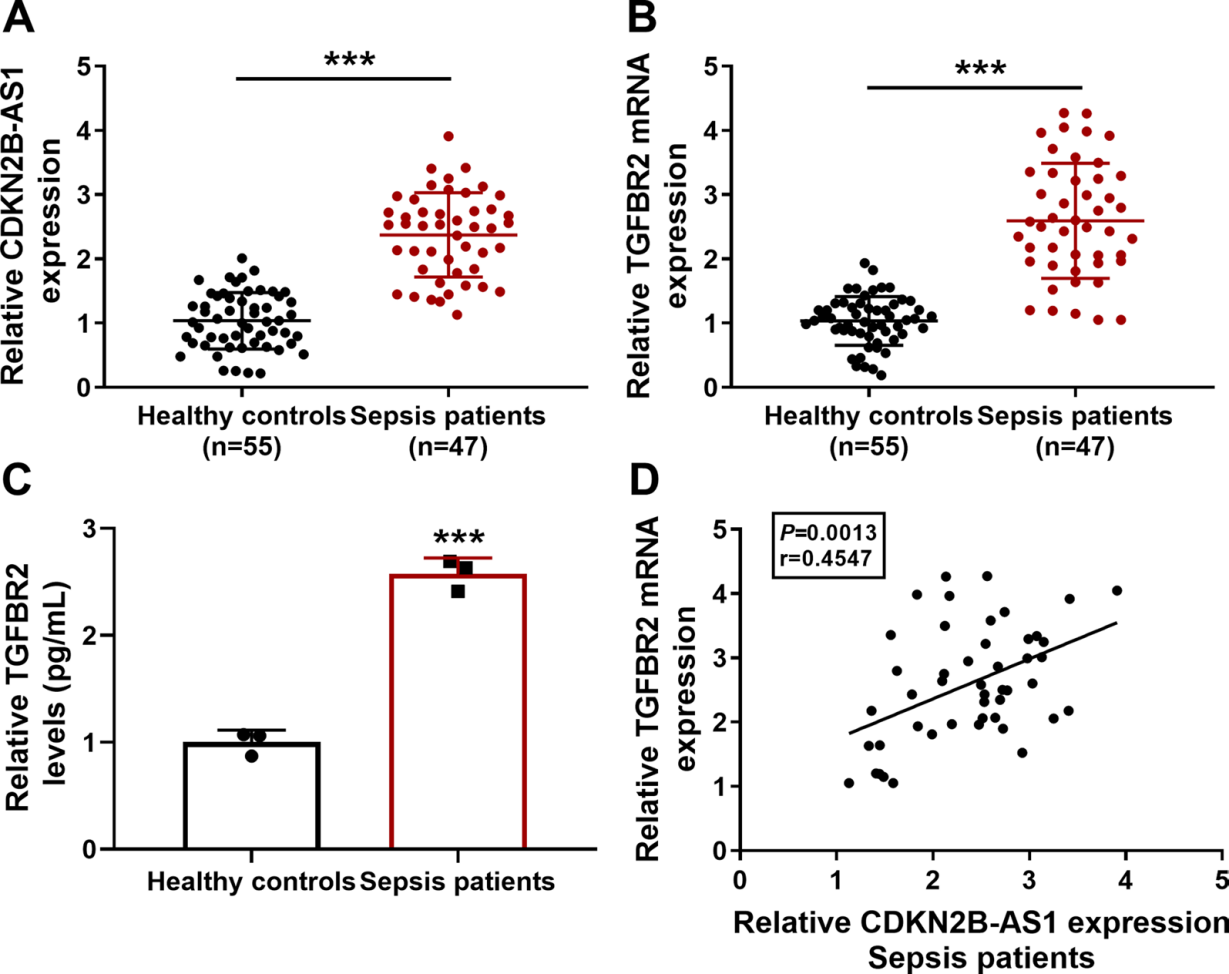
CDKN2B-AS1 and TGFBR2 were overexpressed in sepsis patients. **a**, **b** CDKN2B-AS1 (**a**) and TGFBR2 mRNA (**b**) levels were examined by qRT-PCR in serum samples of 47 sepsis patients and 55 healthy controls. GAPDH was used as an internal control, and the relative expression in sepsis group was compared to the healthy control group (set as 1). (**c**) TGFBR2 protein level was detected by ELISA in 47 sepsis patients compared to 55 healthy controls. (**d**) Pearson’s correlation coefficient was performed for the relation analysis between CDK N2B-AS1 and TGFBR2 in 47 sepsis patients. The number of replicates for each experiment was 3 (n = 3). Student’s *t*-test was used for statistical analysis. ***P < 0.001

The detailed characteristics of sepsis patients and healthy volunteers were shown in Table 2. The demographic information indicated that the mean ages of sepsis patients and healthy controls were 54.3 ± 7.2 years and 53.1 ± 5.3 years, respectively. There were 22 females (47%) and 25 males (53%) among sepsis patients, as well as 21 females (38%) and 34 males (62%) among healthy controls. No significant difference was found in demographic characteristics between sepsis patients and healthy controls. Healthy volunteers were enrolled without obvious abnormality in biochemical indexes. The levels of Scr, CRP and WBC were increased but the level of albumin was decreased in sepsis patients relative to healthy controls (*P* < 0.001). In addition, the median value of APACHE II score was 12.6 ± 3.5 in sepsis patients. Subsequently, the expression levels of CDKN2B-AS1 and TGFBR2 in sepsis patients were determined by qRT-PCR. In comparison with the healthy controls, CDKN2B-AS1 (Fig. 1a) and TGFBR2 mRNA (Fig. 1b) levels were significantly increased in serum samples from sepsis patients. ELISA also indicated that the protein level of TGFBR2 was higher in 47 sepsis patients than that in 55 healthy controls (Fig. 1c). Pearson’s correlation coefficient analysis exhibited that CDKN2B-AS1 level was positively related to TGFBR2 level (*P* = 0.0013, r = 0.4547) in sepsis samples (Fig. 1d). These data revealed that CDKN2B-AS1 and TGFBR2 were aberrantly upregulated in sepsis patients.

**Table 2:** Characteristics of participants

Items	Healthy controls (n = 55)	Sepsis patients (n = 47)	*P* value
Age (years), mean ± SD	53.1 ± 5.3	54.3 ± 7.2	0.336
*Sex, no. (%)*			0.379
Female	21(38%)	22(47%)	
Male	34(62%)	25(53%)	
Smoke, no (%)	19(35%)	21(45%)	0.296
Drink, no (%)	26(47%)	23(49%)	0.867
Scr (mg/dL)	0.8 ± 0.2	1.5 ± 0.4	< 0.001
CRP (mg/L)	3.3 ± 1.9	94.3 ± 42.7	< 0.001
Albumin (g/L)	41.2 ± 3.8	23.5 ± 4.1	< 0.001
WBC (*10^9^ /L)	5.5 ± 1.3	18.6 ± 8.9	< 0.001
APACHE II score	–	12.6 ± 3.5	

SD, standard deviation; Scr, serum creatinine; CRP, C-reactive protein; WBC, white blood cell; APACHE II, acute physiology and chronic health evaluation II

### Knockdown of CDKN2B-AS1 increased cell viability but inhibited apoptosis and inflammation in LPS-treated BEAS-2B cells

**Fig. 2:**
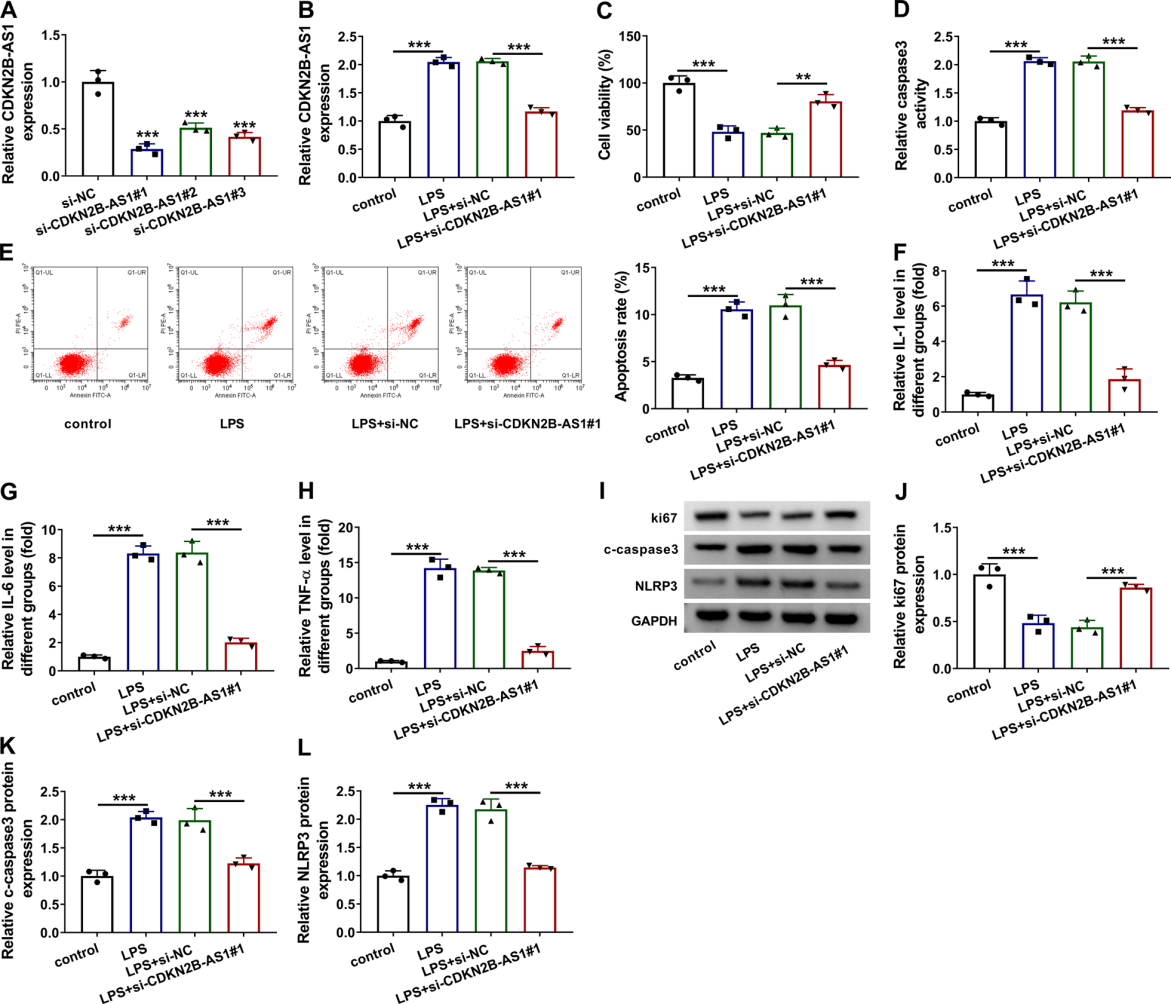
Knockdown of CDKN2B-AS1 increased cell viability but inhibited apoptosis and inflammation in LPS-treated BEAS-2B cells. **a** CDKN2B-AS1 expression was detected using qRT-PCR after transfection of si-NC, si-CDKN2B-AS1#1, si-CDKN2B-AS1#2 or si-CDKN2B-AS1#3. GAPDH was used as an internal control, and the expression of CDKN2B-AS1 in siRNA groups was relative to si-NC group (set as 1). **b** CDKN2B-AS1 detection was performed by qRT-PCR in control, LPS, LPS + si-NC or LPS + si-CDKN2B-AS1#1 group. GAPDH was used as an internal control, and the expression of CDKN2B-AS1 in other three groups was relative to control group (set as 1). **c** Cell viability was measured using CCK-8 assay in the above groups. **d**, **e** Cell apoptosis was assessed through caspase3 activity detection (**d**) and flow cytometry (**e**). **f**–**h** Inflammatory cytokines were determined via ELISA. **i**–**l** Western blot was conducted to analyze the protein levels of ki67, c-caspase3 and NLRP3 in four groups. The number of replicates for each experiment was 3 (n = 3). One-way analysis of variance (ANOVA) followed by Tukey’s test was used for statistical analysis. **P < 0.01, ***P < 0.001

CDKN2B-AS1 level was knocked down by transfection of siRNA. The qRT-PCR results indicated that CDKN2B-AS1 expression of si-CDKN2B-AS1#1 group was lowest among three siRNAs relative to si-NC transfection (Fig. 2a). Thus, si-CDKN2B-AS1#1 was used for the following experiments. Also, transfection of si-CDKN2B-AS1#1 abolished the LPS-induced CDKN2B-AS1 upregulation in BEAS-2B cells (Fig. 2b). Cellular assays demonstrated that LPS treatment resulted in an inhibitory effect on cell viability, but cell viability was then increased after the co-treatment of LPS and si-CDKN2B-AS1#1 (Fig. 2c). The stimulative effects of LPS on caspase3 activity (Fig. 2d) and apoptosis rate (Fig. 2e) were also mitigated by transfection of si-CDKN2B-AS1#1. ELISA was used to assess the inflammatory response. As Fig. 2f–h depicted, CDKN2B-AS1 downregulation repressed the levels of inflammatory cytokines (IL-1, IL-6 and TNF-α) in LPS-treated BEAS-2B cells. Western blot (Fig. 2i) manifested that the protein expression of proliferation marker ki67 was downregulated (Fig. 2j) but the protein levels of apoptosis marker c-caspase3 (Fig. 2k) and NLRP3 inflammasome (Fig. 2l) were upregulated by LPS, whereas these expression changes were all weakened following the introduction of si-CDKN2B-AS1#1. Taken together, the knockdown of CDKN2B-AS1 could ameliorate the LPS-induced lung apoptosis and inflammation.

### Downregulation of TGFBR2 inhibited the LPS-induced injury in BEAS-2B cells

**Fig. 3:**
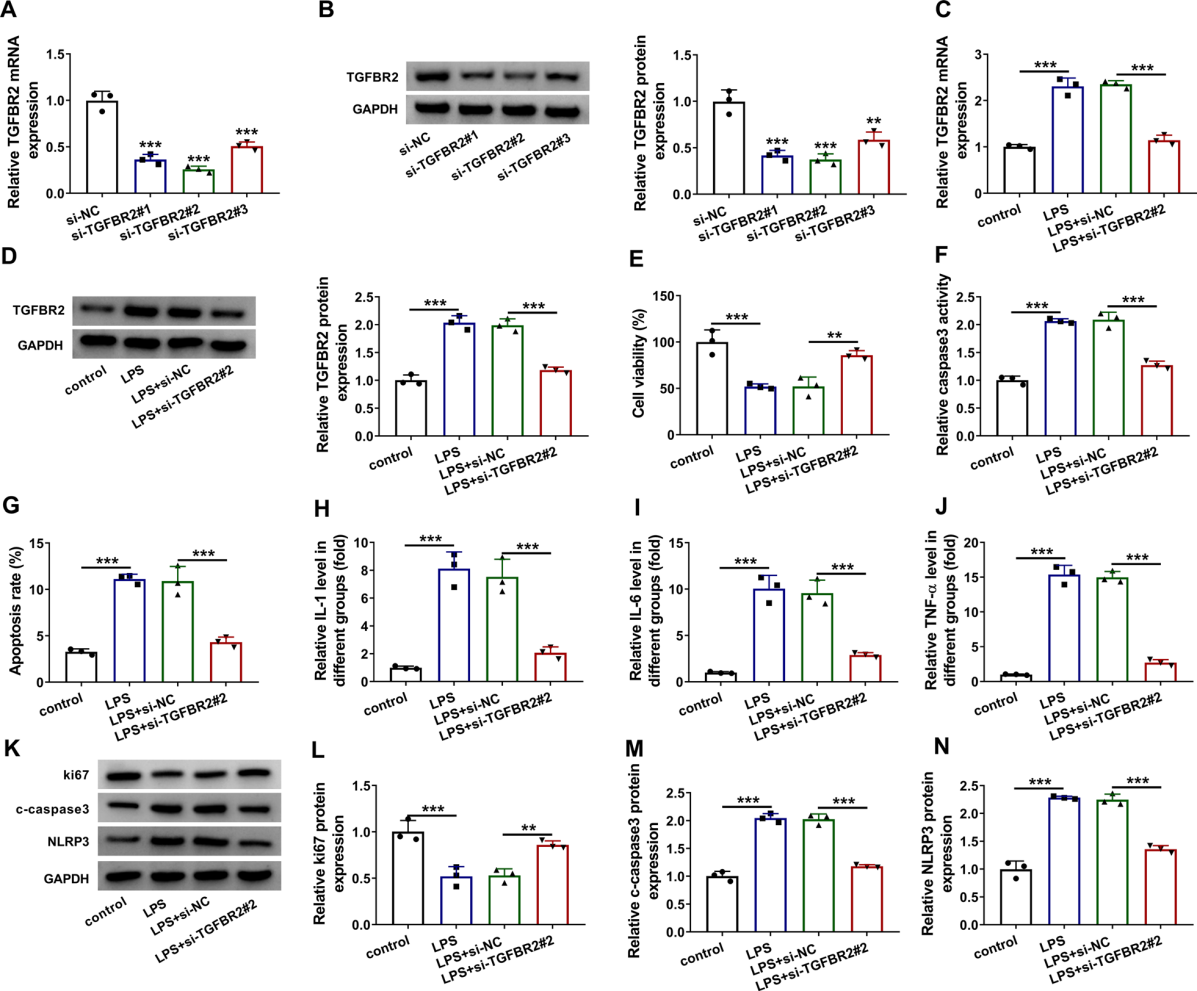
Downregulation of TGFBR2 inhibited the LPS-induced injury in BEAS-2B cells. a, b The qRT-PCR and western blot were used for evaluating the knockdown efficiency of si-TGFBR2#1, si-TGFBR2#2 and si-TGFBR2#3. GAPDH was used as an internal control, and TGFBR2 expression in siRNA groups was relative to si-NC group (set as 1). c, d TGFBR2 mRNA and protein levels were quantified by qRT-PCR and western blot in control, LPS, LPS + si-NC and LPS + si-TGFBR2#2 groups. GAPDH was used as an internal control, and TGFBR2 expression in other three groups was relative to control group (set as 1). e–j CCK-8 assay, caspase3 viability/flow cytometry and ELISA were applied to assess cell viability (e), cell apoptosis (f, g) and inflammatory response (h–j) in control, LPS, LPS + si-NC or LPS + si-TGFBR2#2 group. k–n The detection of ki67, c-caspase3 and NLRP3 was performed by western blot in the above groups. The number of replicates for each experiment was 3 (n = 3). One-way analysis of variance (ANOVA) followed by Tukey’s test was used for statistical analysis. **P < 0.01, ***P < 0.001

The function of TGFBR2 was also explored by siRNA transfection. The results of qRT-PCR and western blot suggested that the knockdown efficiency of si-TGFBR2#2 was better than si-TGFBR2#1 and si-TGFBR2#3 (Fig. 3a, b). Meanwhile, TGFBR2 mRNA and protein levels were reduced in LPS + si-TGFBR2#2 group relative to LPS + si-NC group (Fig. 3c, d). LPS-mediated cell viability suppression (Fig. 3e), apoptosis promotion (Fig. 3f, g) and inflammatory response (Fig. 3h–j) were all attenuated after TGFBR2 expression was downregulated. In addition, knockdown of TGFBR2 upregulated the ki67 protein expression while reduced the c-caspase3 and NLRP3 protein levels in LPS-treated BEAS-2B cells (Fig. 3k–n). Thus, LPS-induced cell injury was also relieved by the downregulation of TGFBR2.

### CDKN2B-AS1 regulated the LPS-induced apoptotic and inflammatory damages via the positive regulation on TGFBR2

**Fig. 4:**
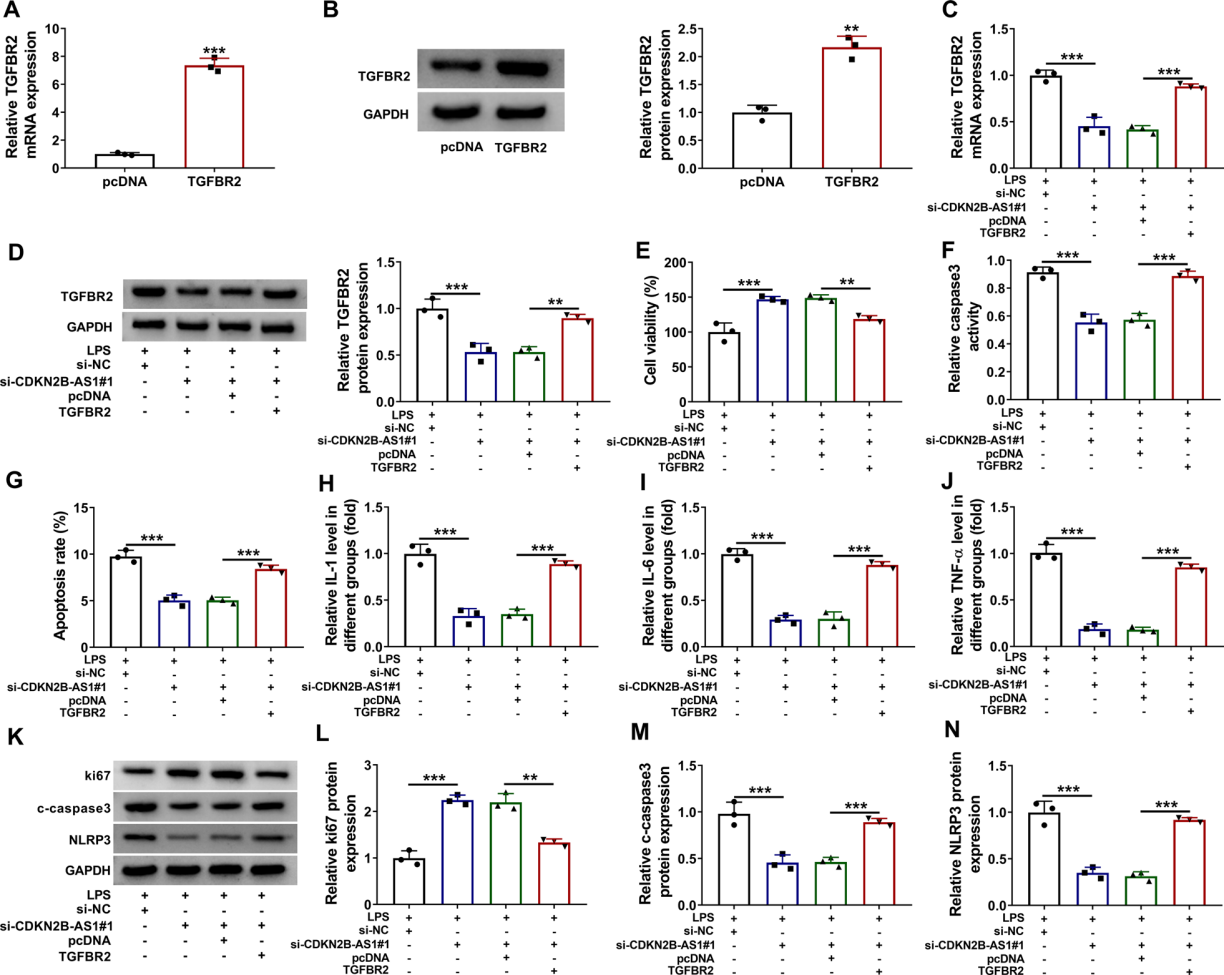
CDKN2B-AS1 regulated the LPS-induced apoptotic and inflammatory damages via the positive regulation on TGFBR2. **a**, **b** The overexpression efficiency of TGFBR2 was analyzed by qRT-PCR and western blot. GAPDH was used as an internal control, and TGFBR2 expression in TGFBR2 group was relative to pcDNA group (set as 1). **c, d** The qRT-PCR and western blot were performed for TGFBR2 expression detection in the following groups: LPS + si-NC, LPS + si-CDKN2B-AS1#1, LPS + si-CDKN2B-AS1#1 + pcDNA, and LPS + si-CDKN2B-AS1#1 + TGFBR2. GAPDH was used as an internal control, and TGFBR2 expression in other three groups was relative to LPS + si-NC group (set as 1). **e**–**j** The measurement of cell viability (**e**), cell apoptosis (**f**, **g**) and inflammatory response (**h–j**) was carried out via CCK-8 assay, caspase3 viability assay/flow cytometry and ELISA in the above groups. **k**–**n** The expression levels of ki67, c-caspase3 and NLRP3 proteins in these groups were examined by western blot. The number of replicates for each experiment was 3 (n = 3). Student’s *t*-test and one-way analysis of variance (ANOVA) followed by Tukey’s test were used for statistical analysis. **P < 0.01, ***P < 0.001

The regulatory relation between CDKN2B-AS1 and TGFBR2 in LPS-induced cell injury was further investigated. The qRT-PCR and western blot assays showed that the mRNA and protein levels of TGFBR2 were significantly increased in TGFBR2 transfection group compared to pcDNA transfection group, which suggested that the overexpression efficiency of TGFBR2 was great (Fig. 4a, b). TGFBR2 mRNA and protein levels were downregulated by si-CDKN2B-AS1 in LPS-treated BEAS-2B cells, while this downregulation was abrogated by TGFBR2 transfection (Fig. 4c, d). The promoting effect of si-CDKN2B-AS1#1 on cell viability (Fig. 4e) but the inhibitory effects on cell apoptosis (Fig. 4f, g) and inflammatory response (Fig. 4h–j) in LPS-treated BEAS-2B cells were partly attenuated after the overexpression of TGFBR2. The same reversal of TGFBR2 transfection was observed on the si-CDKN2B-AS1#1-induced expression changes of ki67, c-caspase3 and NLRP3 protein levels (Fig. 4k–n). These findings suggested that the inhibitory effect of si-CDKN2B-AS1#1 on LPS-induced cell injury was dependent on the downregulation of TGFBR2.

### CDKN2B-AS1 targeted miR-140-5p and TGFBR2 was a downstream gene of miR-140-5p

**Fig. 5:**
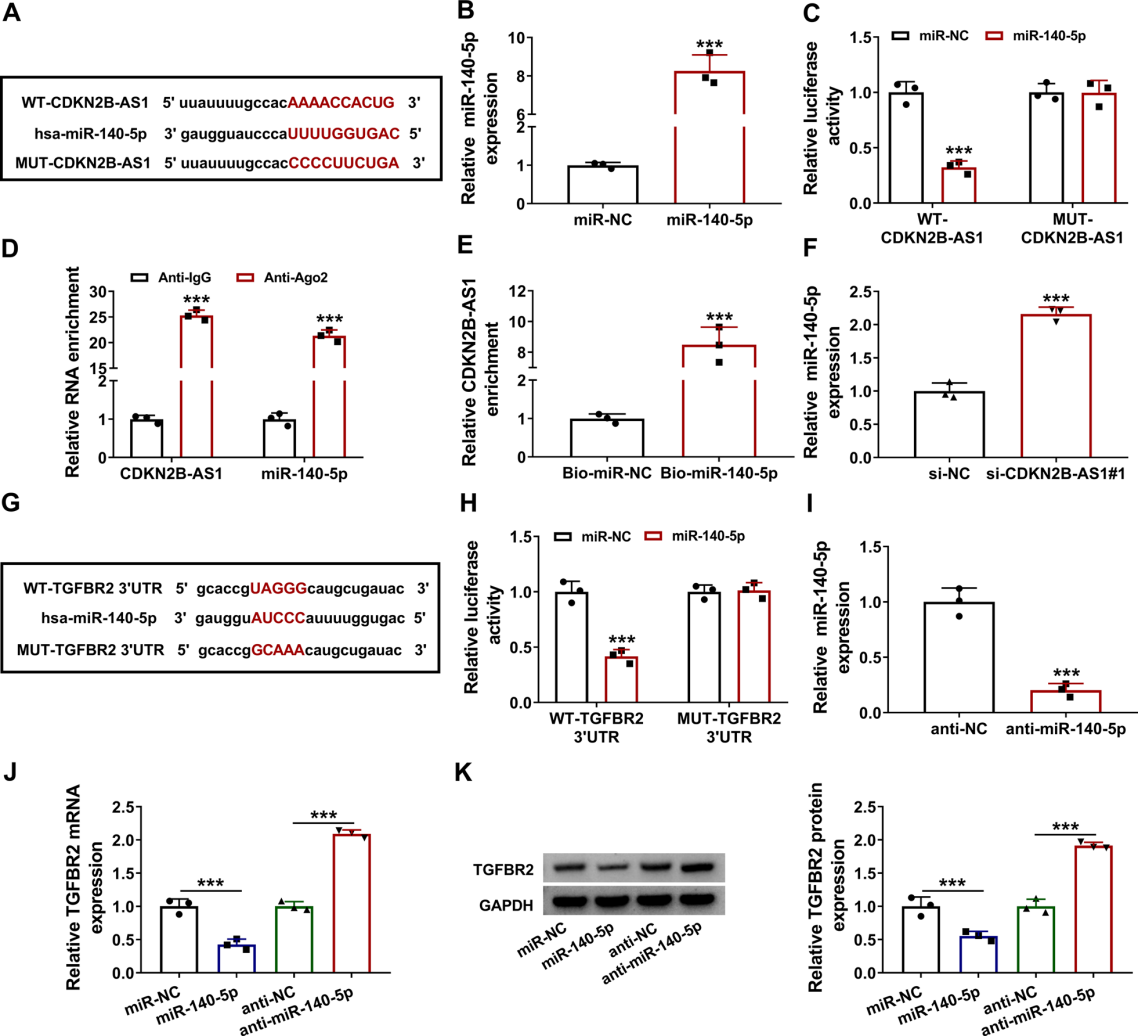
CDKN2B-AS1 targeted miR-140-5p and TGFBR2 was a downstream gene of miR-140-5p. **a** The binding sites between CDKN2B-AS1 and miR-140-5p were predicted by lncBase. **b** The transfection efficiency of miR-140-5p mimic was evaluated by qRT-PCR. U6 was used as an internal control, and miR-140-5p expression in miR-140-5p group was relative to miR-NC group (set as 1). **c–e** The binding between CDKN2B-AS1 and miR-140-5p was affirmed using dual-luciferase reporter assay (**c**), RIP (**d**) and pull-down assay (**e**). **f** The miR-140-5p level was measured in BEAS-2B cells transfected with si-NC or si-CDKN2B-AS1#1. U6 was used as an internal control, and miR-140-5p expression in si-CDKN2B-AS1#1 group was relative to si-NC group (set as 1). **g** The miRWalk software was applied for the bioinformatics analysis between miR-140-5p and TGFBR2. **h** Dual-luciferase reporter assay was conducted to validate the interaction between miR-140-5p and TGFBR2. **i** The miR-140-5p level was examined via qRT-PCR after transfection of anti-NC or anti-miR-140-5p. U6 was used as an internal control, and miR-140-5p expression in anti-miR-140-5p group was relative to anti-NC group (set as 1). **j–k** TGFBR2 mRNA and protein levels were determined through western blot following the transfection of miR-NC, miR-140-5p, anti-NC or anti-miR-140-5p. GAPDH was used as an internal control, and TGFBR2 expression in miR-140-5p or anti-miR-140-5p group was relative to miR-NC or anti-NC group (set as 1). The number of replicates for each experiment was 3 (n = 3). Student’s *t*-test was used for statistical analysis. ***P < 0.001

LncRNAs can regulate the progression of many diseases via acting as the sponges of different miRNAs [28, 29]. The bioinformatics analysis by online lncBase indicated that the sequence of CDKN2B-AS1 contained the complementary sites for miR-140-5p (Fig. 5a). The expression of miR-140-5p was significantly increased by miR-140-5p mimic, contrasted to miR-NC control group (Fig. 5b). Overexpression of miR-140-5p has not affected the relative luciferase activity of MUT-CDKN2B-AS1 plasmid but inhibited that of WT-CDKN2B-AS1 plasmid (Fig. 5c). Thus, CDKN2B could interact with miR-140-5p. RIP assay also exhibited that CDKN2B-AS1 and miR-140-5p were enriched by Ago2 protein (Fig. 5d). Furthermore, CDKN2B-AS1 was pulled down by Bio-miR-140-5p relative to Bio-miR-NC group (Fig. 5e). Moreover, the qRT-PCR showed that knockdown of CDKN2B-AS1 upregulated the level of miR-140-5p in BEAS-2B cells (Fig. 5f). Meanwhile, miRWalk software predicted there were the binding sites between TGFBR2 3’UTR and miR-140-5p sequences (Fig. 5g). Dual-luciferase reporter assay manifested that miR-140-5p upregulation could reduce the luciferase activity in WT-TGFBR2 3’UTR group but failed to affect that in MUT-TGFBR2 3’UTR group (Fig. 5h). The anti-miR-140-5p-mediated expression inhibition of miR-140-5p was successful in BEAS-2B cells (Fig. 5i). The qRT-PCR and western blot results manifested that overexpression of miR-140-5p repressed the mRNA and protein levels of TGFBR2, while miR-140-5p inhibitor induced the upregulation of TGFBR2 (Fig. 5j, k). The above evidence suggested that CDKN2B-AS1 served as a sponge of miR-140-5p and TGFBR2 was a target of miR-140-5p.

### CDKN2B-AS1 promoted the LPS-induced cell injury via the miR-140-5p/TGFBR2/Smad3 axis

**Fig. 6:**
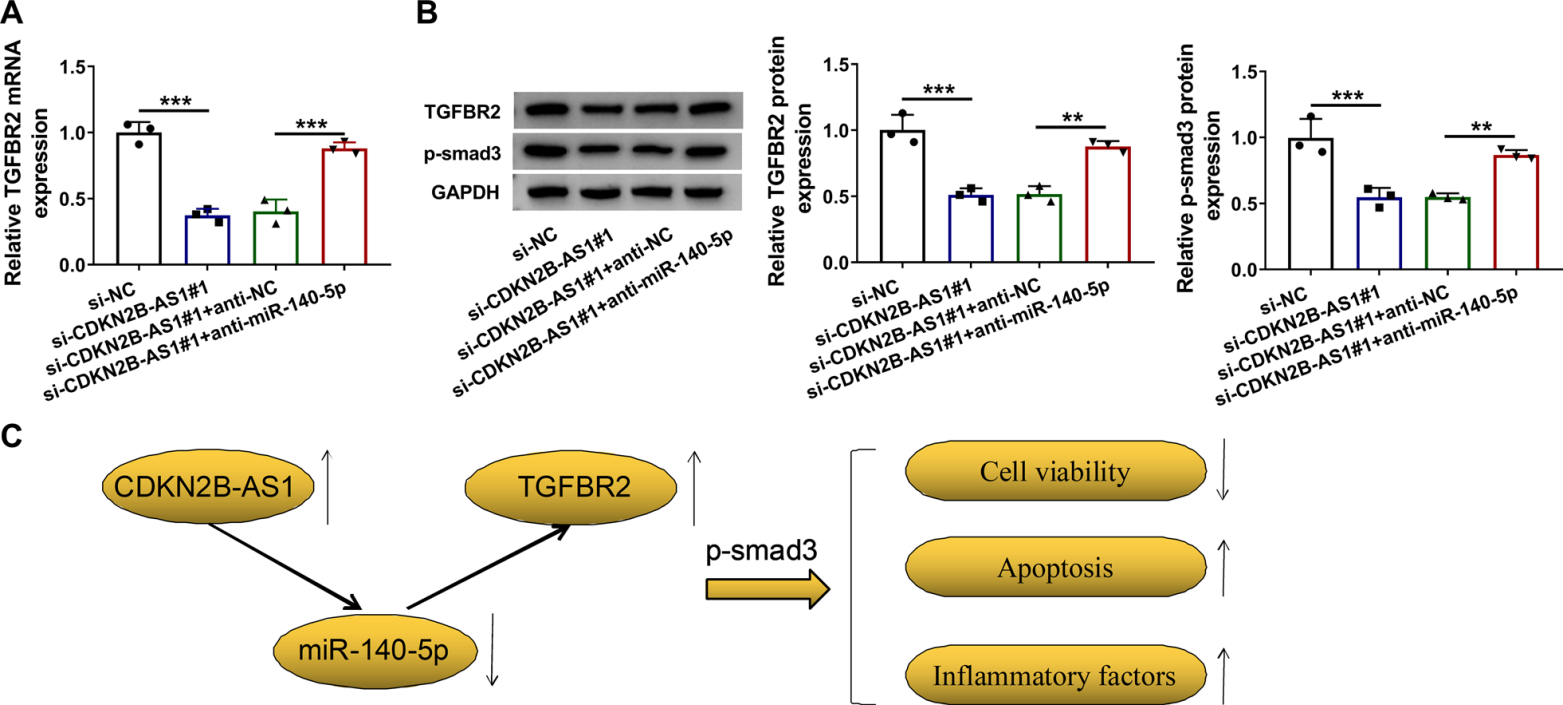
CDKN2B-AS1 promoted the LPS-induced cell injury via the miR-140-5p/TGFBR2/Smad3 axis. **a**, **b** The qRT-PCR and western blot were respectively performed to detect TGFBR2 mRNA expression (**a**) and TGFBR2/Smad3 protein levels (**b**) after si-NC, si-CDKN2B-AS1#1, si-CDKN2B-AS1#1 + anti-NC or si-CDKN2B-AS1#1 + anti-miR-140-5p transfection. GAPDH was used as an internal control, and the protein expression in other three groups was relative to si-NC group (set as 1). **c** The graphical summary of the CDKN2B-AS1/miR-140-5p/TGFBR2/Smad3 axis in the regulation of LPS-induced cell damages. The number of replicates for each experiment was 3 (n = 3). One-way analysis of variance (ANOVA) followed by Tukey’s test was used for statistical analysis. **P < 0.01, ***P < 0.001

To analyze whether the regulation of CDKN2B-AS1 on the TGFBR2 level was related to miR-140-5p, we determined the expression of TGFBR2 after the reverted transfection (si-NC, si-CDKN2B-AS1#1, si-CDKN2B-AS1#1 + anti-NC or si-CDKN2B-AS1#1 + anti-miR-140-5p). As the qRT-PCR result in Fig. 6a, anti-miR-140-5p reversed the si-CDKN2B-AS1#1-induced downregulation of TGFBR2 mRNA expression. Western blot demonstrated that knockdown of CDKN2B-AS1 suppressed the protein levels of TGFBR2 and phosphorylated Smad3 via upregulating the level of miR-140-5p, suggesting that CDKN2B-AS1 could activate the TGFBR2/smad3 pathway (Fig. 6b). Altogether, CDKN2B-AS1 acted as a sponge of miR-140-5p to regulate the downstream TGFBR2/Smad3 signals, consequently inhibiting cell viability but promoting cell apoptosis and inflammation in LPS-treated lung epithelial cells (Fig. 6c).

## Discussion

ALI is a common sepsis-related complication. The present study suggested that CDKN2B-AS1 enhanced cell apoptosis and inflammation in LPS-treated lung epithelial cells by regulating the miR-140-5p/TGFBR2/Smad3 signal network. These findings might lay a great foundation for the research of molecular mechanism in sepsis-related ALI.

The issued studies have shown that lncRNAs were implicated in the regulation of sepsis. For instance, Wu et al. discovered that KCNQ1OT1 reduced the sepsis-induced myocardial injury through the upregulation of XIAP by serving as a sponge of miR-192-5p [30]. HOTAIR was reported to attenuate acute kidney injury in sepsis rats by targeting the miR-34a/Bcl-2 axis [31]. MALAT1 was found to aggravate the sepsis-induced cardiac inflammation via regulating the miR-150-5p/NF-κB signal pathway [32]. Additionally, CASC9 and TUG1 alleviated the sepsis-induced ALI via affecting the miR-195-5p/PDK4 axis [33] and miR-34b-5p/GAB1 axis [34]. Liang et al. unraveled that MALAT1 enhanced the LPS-induced inflammatory response in lung epithelial cells via increasing the expression of MyD88 by sponging miR-149 [35]. Herein, we found that CDKN2B-AS1 expression was upregulated in serum samples from sepsis patients. The functional analysis exhibited that the downregulation of CDKN2B-AS1 protected BEAS-2B cells against the LPS-induced cell viability inhibition and apoptotic or inflammatory injury. These data confirmed that CDKN2B-AS1 aggravated the LPS-induced cell damages in lung epithelial cells, implying that CDKN2B-AS1 might be involved in the sepsis-induced ALI. The potential role of CDKN2B-AS1 in sepsis-related ALI still needs further exploration.

TGFBR2 has attenuated the miR-145-mediated inhibition of sepsis progression in LPS-treated human umbilical vein endothelial cells [36]. Our data showed that TGFBR2 was highly expressed in sepsis patients. Knockdown of TGFBR2 promoted cell viability but reduced cell apoptosis and inflammation in LPS-treated BEAS-2B cells, indicating that TGFB2 might be associated with the sepsis-related lung injury. More interestingly, TGFBR2 overexpression abrogated the protective function of CDKN2B-AS1 knockdown in LPS-treated lung cells. Therefore, the effect of CDKN2B-AS1 on LPS-mediated lung cell dysfunction was achieved partly by the upregulation of TGFBR2.

LncRNA/miRNA/mRNA axis has been found in various kinds of human diseases [37–39]. Our bioinformatical prediction displayed that miR-140-5p might be a target for CDKN2B-AS1. The interaction between CDKN2B-AS1 and miR-140-5p was further validated by dual-luciferase reporter, RIP and pull-down assays. CDKN2B-AS1 was found to have the negative regulation of miR-140-5p expression, indicating the sponge effect of CDKN2B-AS1 on miR-140-5p. Meanwhile, the online prediction revealed the binding sites between miR-140-5p and TGFBR2. Dual-luciferase reporter assay also affirmed that miR-140-5p bound to TGFBR2 3’UTR, and the expression detection demonstrated that miR-140-5p could directly downregulate the TGFBR2 level. Furthermore, our expression analysis revealed that CDKN2B-AS1 induced the indirect upregulation of TGFBR2 by targeting miR-140-5p. Moreover, CDKN2B-AS1/miR-140-5p axis could regulate the p-smad3 protein expression. Smad3 has been identified as a downstream gene of TGFBR2 [40]. Thus, we considered that CDKN2B-AS1/miR-140-5p axis activated the smad3 signal via upregulating the TGFBR2 expression.

However, the current study has some limitations. Firstly, all experiments were performed in a single bronchial epithelial cell line. The further research in primary cells or other small airway epithelial cells may provide support for the conclusion. Secondly, it is uncertain whether LPS-treated BEAS-2B is an applicable cell model of sepsis-induced ALI. The present findings only suggested that CDKN2B-AS1 regulated the LPS-induced apoptotic and inflammatory damages in BEAS-2B cells. Finally, all assays were conducted in vitro. In vivo assay is necessary to affirm the conclusion in future.

## Conclusions

In conclusion, this study suggested that CDKN2B-AS1 contributed to LPS-mediated lung dysfunction in lung epithelial cells by targeting miR-140-5p to regulate the TGFBR2/Smad3 axis. CDKN2B-AS1 has the potential to act as a therapeutic target for sepsis-related ALI.

## Supplementary Information

**Additional file 1**. The original protein images for western blot assay.

## Data Availability

The data sets used and/or analyzed during the current study are available from the corresponding author on reasonable request.

## Abbreviations

ALI: Acute lung injury
lncRNAs: Long non-coding RNAs
CDKN2B-AS1L: Cyclin-dependent kinase inhibitor 2B antisense RNA 1
LPS: Lipopolysaccharide
TGFBR2: Transforming growth Factor Beta Receptor II
qRT-PCR: Quantitative real-time polymerase chain reaction
CCK-8: Cell counting Kit-8
RIP: RNA immunoprecipitation
ELISA: Enzyme-linked immunosorbent assay
