# Supplementary material for: Long non-coding RNA CDKN2B-AS1 enhances LPS-induced apoptotic and inflammatory damages in human lung epithelial cells via regulating the miR-140-5p/TGFBR2/Smad3 signal network
Source: BMC Pulm Med. 2021 Jun 14;21:200. doi: 10.1186/s12890-021-01561-z (PMC8201744; doi:10.1186/s12890-021-01561-z)
Supplement: Supplementary file 1 — Additional file 1. The original protein images for western blot assay. [file 12890_2021_1561_MOESM1_ESM.pdf]

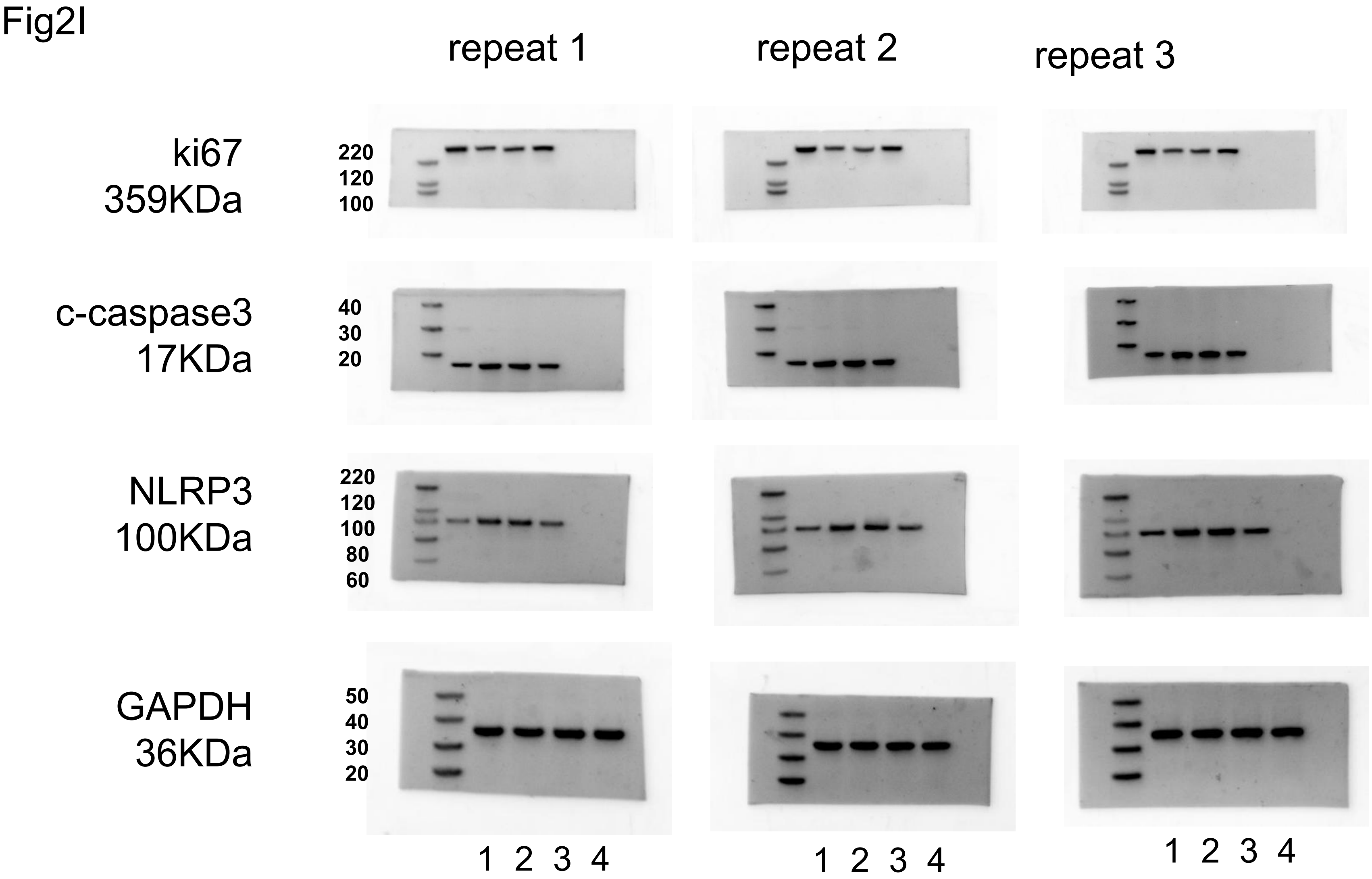

2I: The protein levels of ki67, c-caspase3 and NLRP3 in four groups （1:Control; 2:LPS; 3:LPS+si-NC; 4:LPS+si-CDKN2B-AS1#1;）were detected by western blot

Fig3B

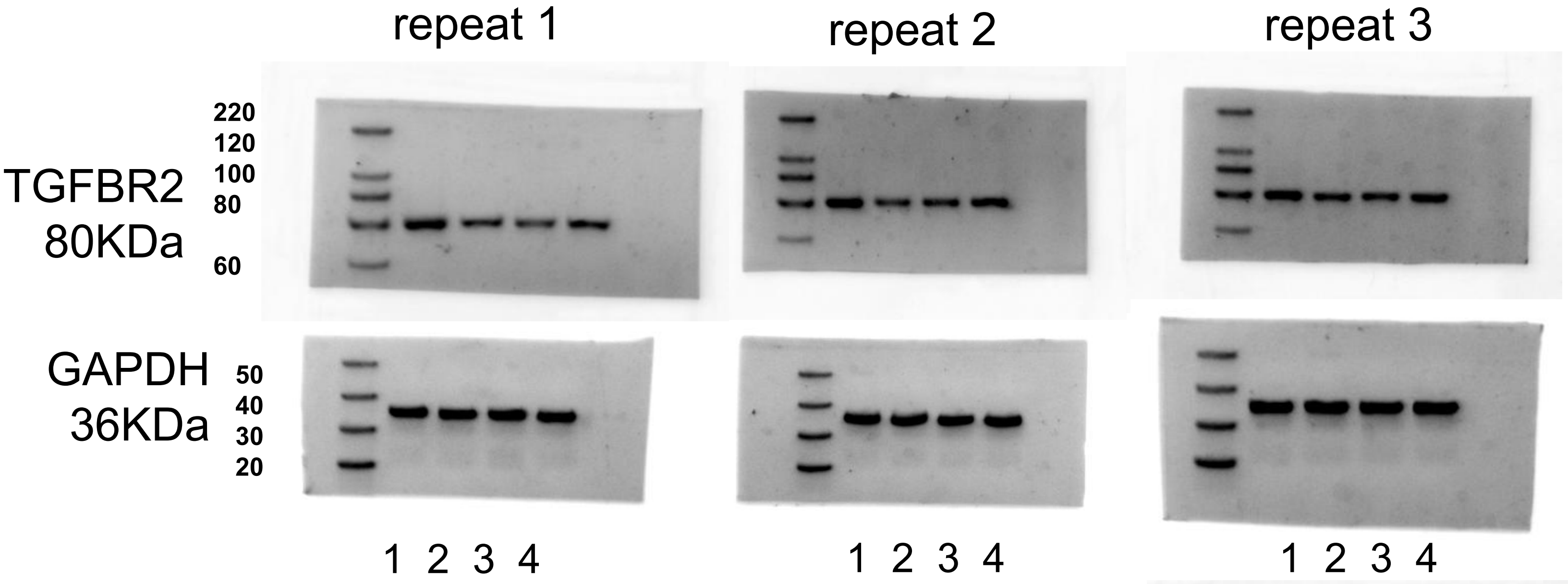

Fig3D

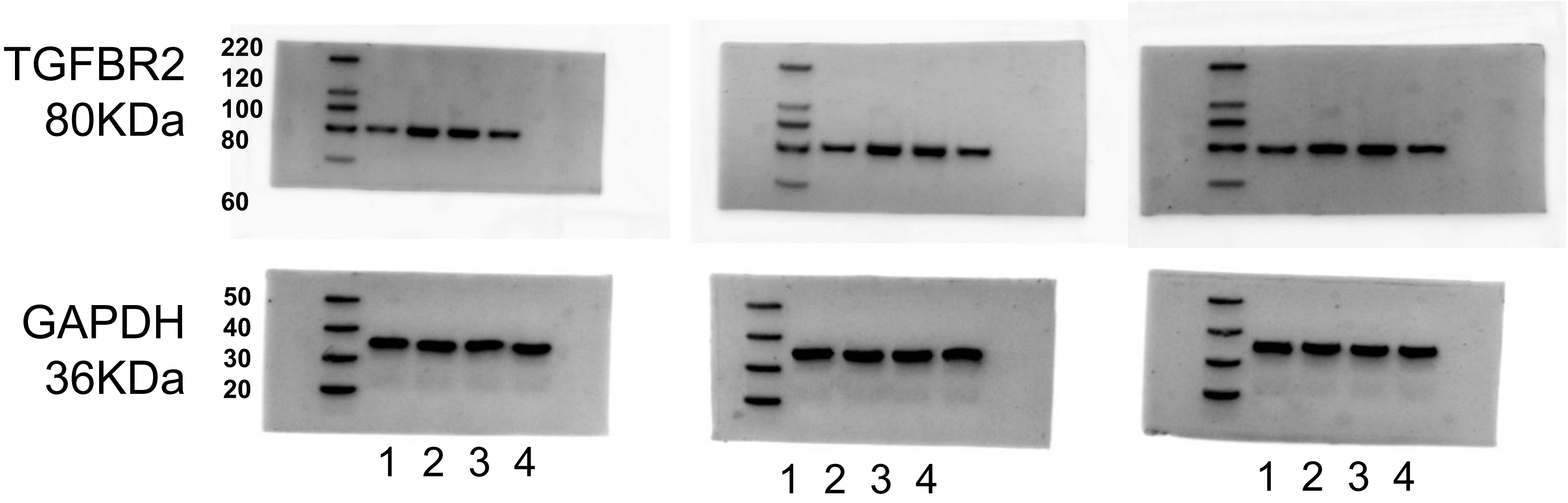

3B: The protein levels of TGFBR2 in four groups （1:si-NC; 2:si-TGFBR2#1; 3:si-TGFBR2#2; 4:si-TGFBR2#3;）were detected by western blot

3D: The protein levels of TGFBR2 in four groups （1:Control; 2:LPS; 3:LPS+si-NC; 4:LPS+si-TGFBR2#2;）were detected by western blot

Fig3K

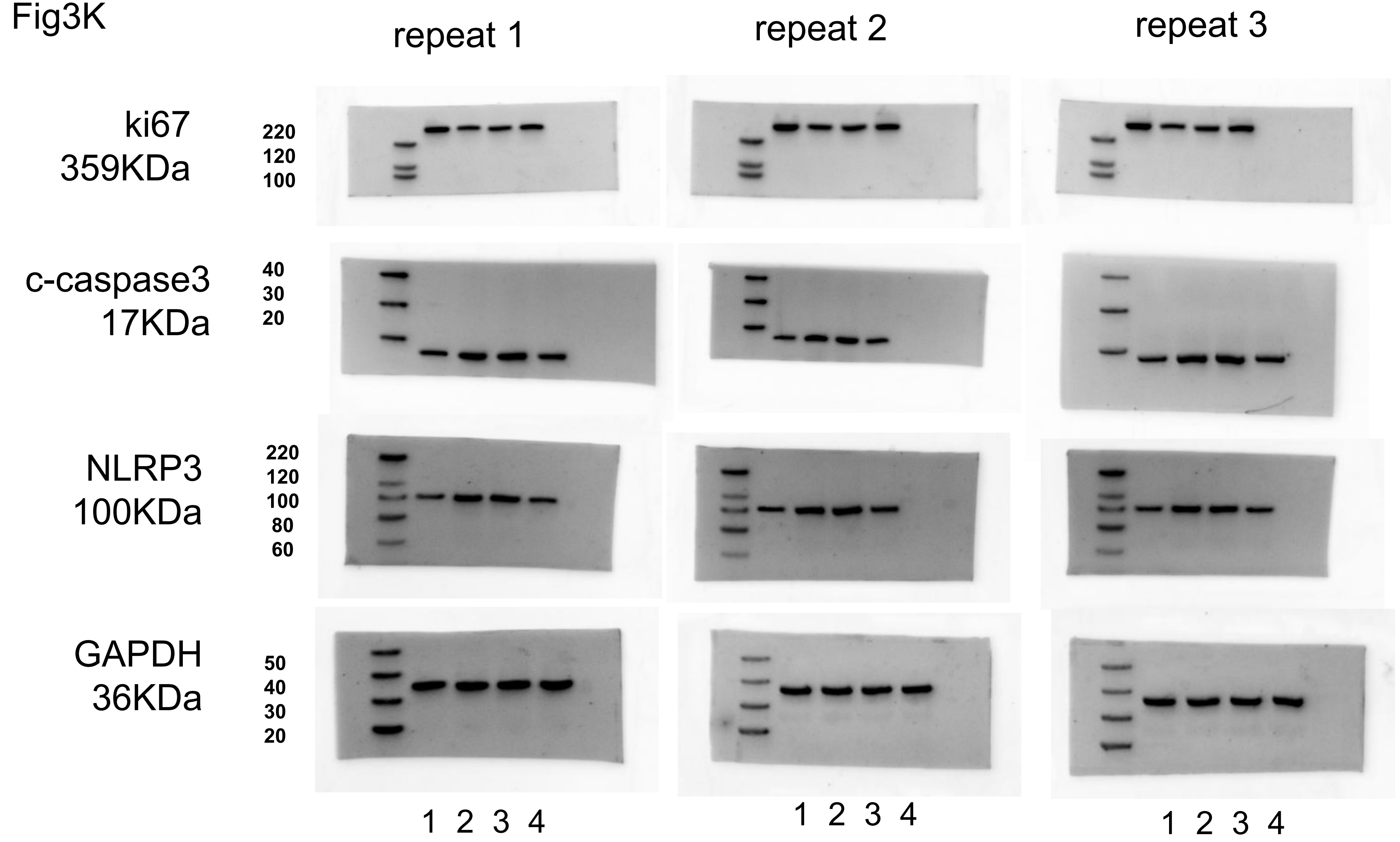

3K: The protein levels of ki67, c-caspase3 and NLRP3 in four groups（1:Control; 2:LPS; 3:LPS+si-NC; 4:LPS+si-TGFBR2#2;）were detected by western blot

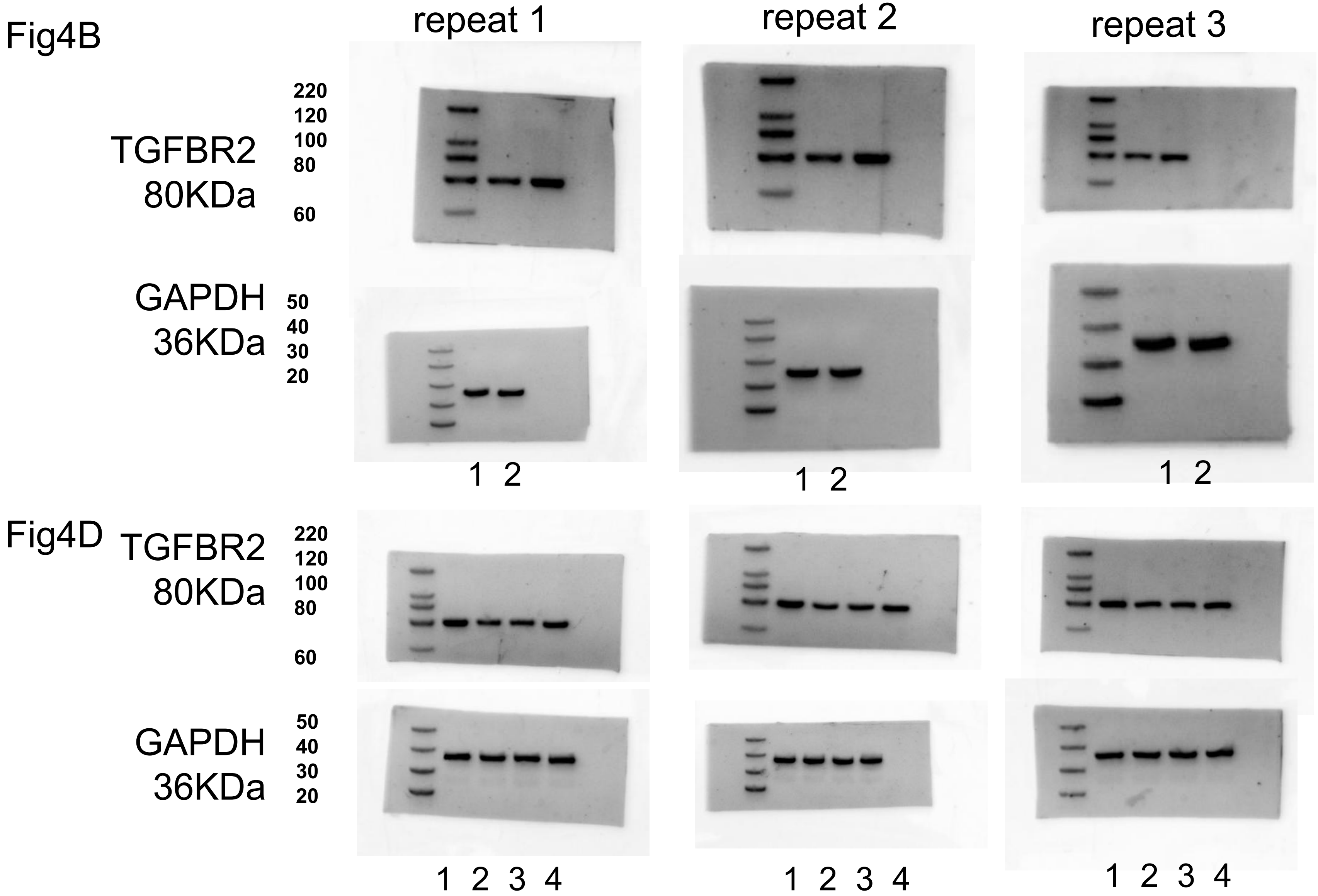

4B: The protein levels of TGFBR2 in two groups （1:pcDNA; 2:TGFBR2;） were detected by western blot

4D: The protein levels of TGFBR2 in four groups （1: LPS+si-NC; 2:LPS+si-CDKN2B-AS1#1; 3:LPS+si-CDKN2B-AS1#1+pcDNA; 4:LPS+si-CDKN2B-AS1#1+TGFBR2;）were detected by western blot

Fig4K

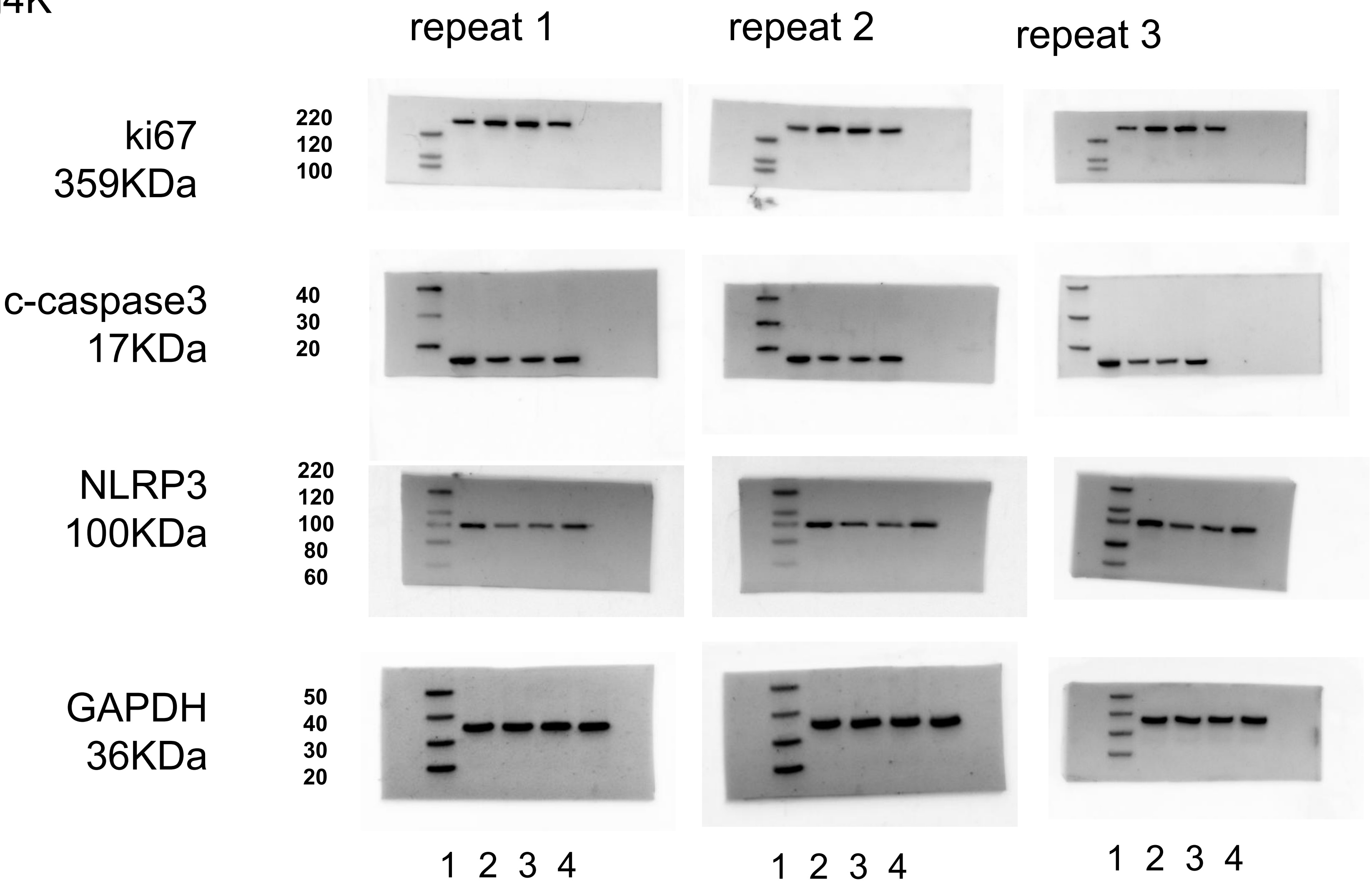

4K: The protein levels of ki67, c-caspase3 and NLRP3 in four groups （1:LPS+si-NC; 2:LPS+si-CDKN2B-AS1#1; 3:LPS+si-CDKN2B-AS1#1+pcDNA; 4:LPS+si-CDKN2B-AS1#1+TGFBR2;） were detected by western blot

Fig5K

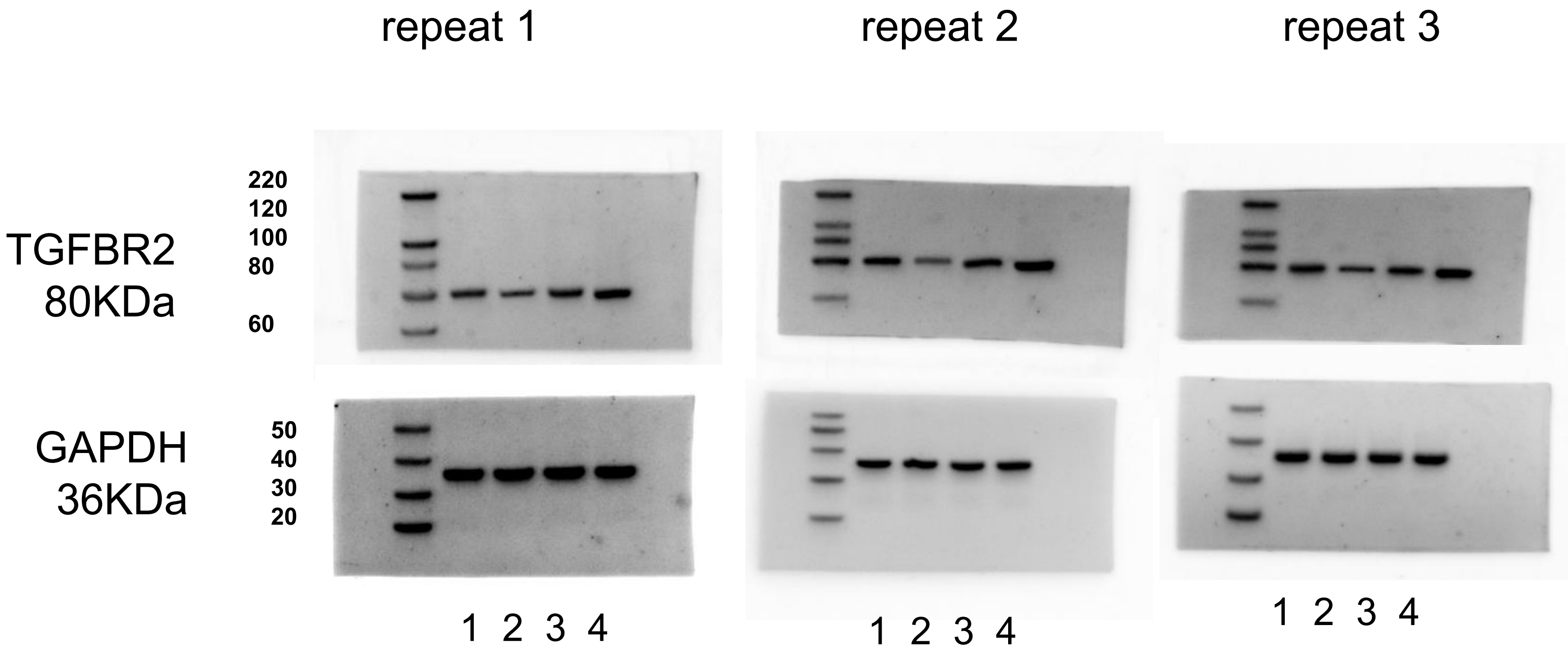

5K: The protein levels of TGFBR2 in four groups （1:miR-NC; 2:miR-140-5p; 3:anti-NC; 4:anti-miR-140-5p;）were detected by western blot

Fig6B

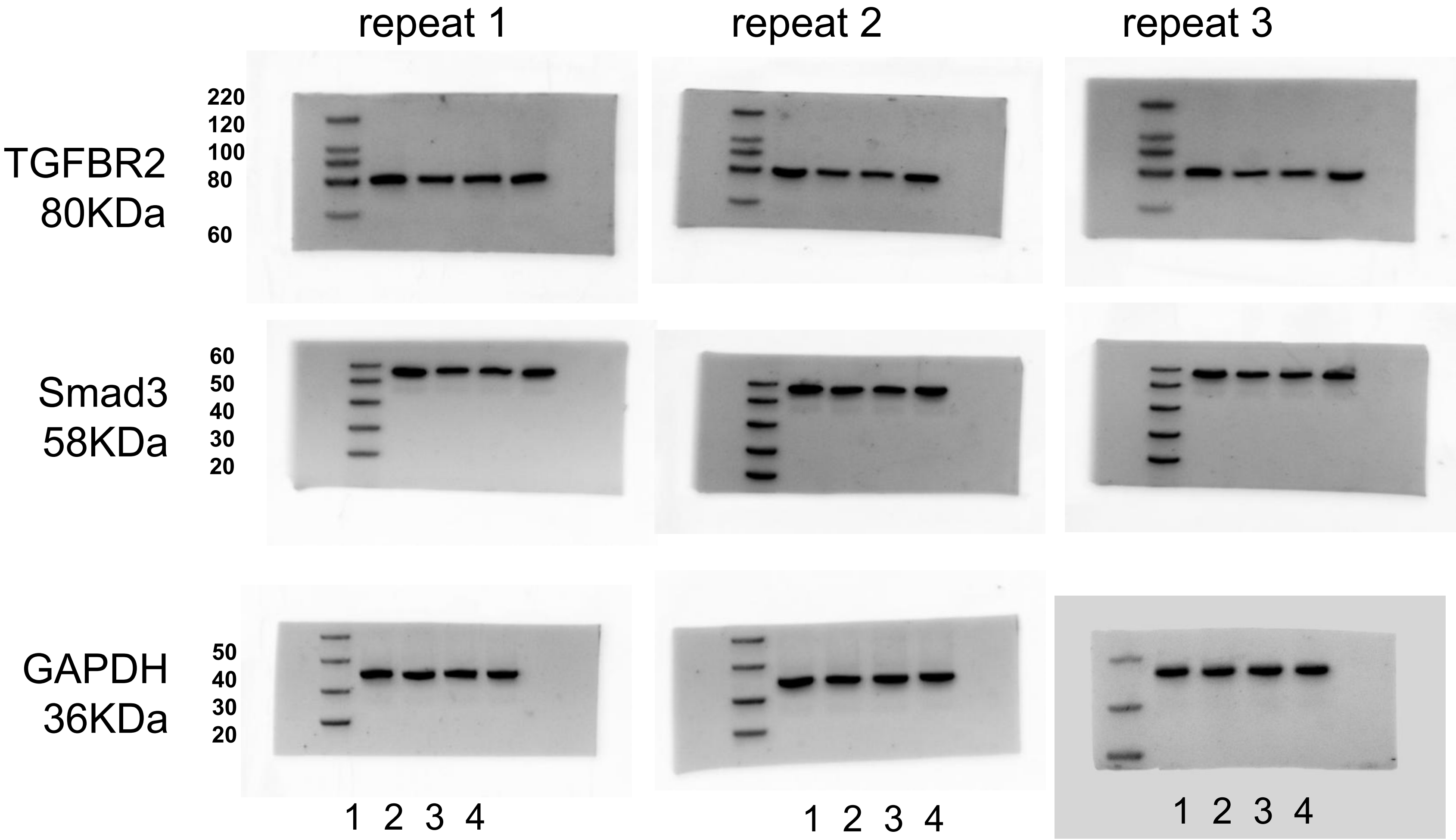

6B: The protein levels of TGFBR2,Smad3 in four groups （1:si-NC; 2:si-CDKN2B-AS1#1; 3:si-CDKN2B-AS1#1+anti-NC; 4:si-CDKN2B-AS1#1+anti-miR-140-5p;） were detected by western blot
